# Nodal disease predicts recurrence whereas other traditional factors affect survival in a cohort of South African patients with differentiated thyroid carcinoma

**DOI:** 10.1186/s41199-018-0037-5

**Published:** 2018-11-19

**Authors:** B. Robertson, M. Parker, L. Shepherd, E. Panieri, L. Cairncross, F. Malherbe, IL. Ross, F. Omar, A. Hunter

**Affiliations:** 1 Department of Radiation Oncology, Cape Town, South Africa; 2 Groote Schuur Hospital, Cape Town, South Africa; 3 University of Cape Town, Cape Town, South Africa; 4 Department of Surgery, Cape Town, South Africa; 5 Department of Medicine, Cape Town, South Africa; 6 Division of Chemical Pathology, Cape Town, South Africa

**Keywords:** Differentiated thyroid carcinoma, South Africa, Prognostic factors, Survival

## Abstract

**Background and aim:**

Information on patients with differentiated thyroid carcinoma in South Africa is limited. The objective of this study was to review demographics and tumour characteristics in a cohort of patients with differentiated thyroid carcinoma, presenting to Groote Schuur Hospital and evaluate risk factors for recurrence and survival.

**Patients and methodology:**

Retrospective demographic and clinical data were collected on all patients referred between January 2003 and December 2013. Prognostic factors for recurrence free survival and cancer specific survival were assessed using univariate and multivariate analyses.

**Results:**

The total number of patients was 231.The median age at presentation was 44 years and 82% were female patients. The pathological sub-types were papillary (60.6%), follicular (38.9%) and poorly differentiated (0.5%). Total thyroidectomy was performed in 191 patients and 30 patients required neck dissections. A total of 171 (74%) patients received ^131^Iodine. The recurrence free and cause specific survival rates at 10 years were 83 and 91%, respectively. Nodal disease at presentation was the only significant risk factor for recurrence (*p* <  0.001) on multivariate analysis. Significant risk factors for cause specific mortality were age ≥ 45 years (*p* = 0.006), follicular pathology (*p* = 0.004), extra-thyroid extension (*p* = 0.013) and residual tumour (*p* = 0.004).

**Conclusions:**

Consistent with international trends, patients with differentiated thyroid carcinoma treated at Groote Schuur Hospital had a favourable prognosis. The known risk factors associated with recurrence and survival in this South African cohort were consistent with those reported in developed countries.

## Introduction

Differentiated thyroid carcinoma (DTC), although rare, is the most common endocrine tumour. The disease is more frequently observed in women with a ratio of approximately 2.5:1 [1–3]. The National Cancer Registry of South Africa in 2010 reported that the age standardised incidence rate per 100,000 was 1.13 in females and 0.45 in males and DTC accounted for 0.95 and 0.32% of all cancers in women and men, respectively [3].

Overall, the prognosis is favourable; however, certain features, such as older age, follicular sub-type, larger tumour size, extra-thyroid extension, nodal involvement and metastases are related to poorer outcome [4–9].

There is limited published information on South African patients with differentiated thyroid carcinoma. It is assumed that risk factors that affect prognosis and survival are comparable to those observed in developed countries where most treatment protocols are written. However, it is important to confirm this hypothesis and assess if protocols written in first world countries are applicable to countries in which health resources are limited. In the current study, patient demographics and tumour characteristics in a cohort of patients referred to Groote Schuur Hospital were examined and known prognostic factors were evaluated for an effect on survival and recurrence. These results were compared to outcomes published from centres in the developed world.

## Patients and methodology

### Data collection

Groote Schuur Hospital is an academic hospital associated with the University of Cape Town and serves as a referral centre for the majority of the approximately 4,000,000 people, living in the Cape Town metropolitan area.

Retrospective data were collected from hospital records on all patients with differentiated thyroid carcinoma referred to our unit from January 2003 to December 2013, inclusive. Demographic data, pathology and risk factors for recurrence were recorded. For the purposes of this audit, we used the current British Thyroid Association Guidelines published in 2014 to specify the post-operative risk group for recurrence [10]. Stage at presentation was determined using the American Joint Committee on Cancer (AJCC) staging manual [11, 12]. The extent of surgery, use of ^131^Iodine (^131^I), other treatment modalities and responses were also documented.

### Treatment and follow up

Patients were assessed by a multidisciplinary team, comprising specialists from Endocrine Surgery, Radiation Oncology, Nuclear Medicine, Endocrinology and Anatomical Pathology. The British Thyroid Association guidelines published initially in 2002 [13], and updated in 2007 [14], were the basis for our approach to treatment.

In brief, for patients with a tumour size of ≤1 cm and no associated poor prognostic factors, a lobectomy only was performed. For patients with a tumour size of > 1 cm, multifocal tumours and for smaller tumours with risk factors, a total thyroidectomy was performed. Central compartment neck dissections were not routinely performed, however, any neck nodes suspicious for malignancy were removed at surgery. Patients presenting with metastatic cervical nodes outside the central compartment had the relevant node dissection at the time of total thyroidectomy. After total thyroidectomy, ^131^I for ablation was administered and a post-treatment diagnostic scan was performed. Patients who were treated with ^131^I ablation had a follow-up ^123^I diagnostic scan six to twelve months thereafter, with simultaneous measurements of thyroglobulin and thyroglobulin antibody levels. If there was persistent uptake on the diagnostic scan, therapeutic ^131^I with higher activity was administered. Patients presenting with metastatic disease with Eastern Cooperative Oncology Group Performance Status (ECOG PS) 0–2 were treated with total thyroidectomy and a combination of external beam radiation. In addition, repeated doses of ^131^I, were administered provided that there was persistent iodine avidity, decreasing thyroglobulin levels and expected clinical benefit. On the other hand, patients with persistent or recurrent disease in the neck that was deemed unresectable and not iodine avid, were treated with external beam radiation.

Once the initial treatment was completed, all patients were prescribed thyroxine for thyroid stimulating hormone (TSH) suppression and were followed up clinically. Patients were also monitored for recurrence using thyroglobulin and thyroglobulin antibody levels. Elevated levels prompted further imaging with ultrasound of the neck, and/or ^123^I diagnostic scans to confirm recurrence. Recurrences were assessed to determine suitability for resection, ^131^I or external beam radiation.

### Statistical analysis

Recurrence free survival (RFS) was calculated from the date of initial surgery to the first recorded date of recurrence. Cause specific survival (CSS) and overall survival (OS) were calculated from the date of surgery to the date of death. For the nine patients (4%) who did not have surgery, survival was calculated from the date of diagnosis to death. The time-interval between the date of diagnosis and the date of surgery did not differ by more than six weeks. For overall survival, death from any cause was included and for CSS, thyroid cancer-related death, was included. Patients lost to follow-up were censored at the date of the last recorded clinic visit or last contact with the patient.

All statistical analyses were performed using SPSS (Statistical Package for Social Sciences) version 23 (SPSS, Chicago, IL, USA). Survival (RFS, CSS and OS) analysis was completed using the Kaplan-Meier method [15] and survival curves were compared using the log-rank test. A *p*-value of less than 0.05 was accepted as statistically significant and reported *p*-values were not adjusted for multiple testing.

The influence of potential prognostic factors on RFS and CSS was initially investigated using Log-Rank (Mantel-Cox) univariate analysis. Prognostic factors included age, gender, primary tumour stage (T), extra-thyroid extension, pathology, nodal status, residual tumour, metastases and post-operative risk stratification for recurrence. Variables that were identified as significantly associated with survival on univariate analysis were entered into a Cox proportional hazards regression model for multivariate analysis. The backward-stepwise likelihood ratio (LR) procedure for Cox regression was performed and results were reported as relative risk ratios (hazard ratios: HR) and 95% confidence intervals (CI). In the multivariate analyses, the group of patients with a missing value (unknown) for the prognostic factor evaluated was included with the group that showed a similar survival for that factor. Survival curves where the unknown values were either included or excluded were compared and no significant difference was observed.

## Results

During the assessment period, 231 patients were referred to our unit. The baseline patient and tumour characteristics, as well as staging and initial treatment, are shown in Table 1. There were 189 (82%) females with a median age of 44 years and an interquartile range (IQR) of 33–56 years. The median age for male patients was 53 (IQR 36–64) years. Seventeen patients (7%) had an associated cancer that either antedated or occurred subsequently to the development of thyroid cancer, with the most common primary sites being colorectal and breast. Only four patients had significant risk factors for developing thyroid cancer, with three having a positive family history and one patient who underwent whole body radiation for Hodgkin’s lymphoma.

**Table 1:** Patient and tumour characteristics, treatment details

Characteristic	N (%)
Gender	
Male	42 (18)
Female	189 (82)
Age (years)	
Median (range)	44 (9–89)
Pathology	
Papillary	140 (60.6)
Follicular	90 (38.9)
Poorly differentiated	1 (0.5)
Tumour size	
≤ 1 cm	42 (18.2)
1–4 cm	105 (45.5)
> 4 cm	56 (24.2)
Unknown	28 (12.1)
T stage	
pTX	20 (8.7)
pT0	1 (0.4)
pT1	53 (22.9)
pT2	79 (34.2)
pT3	53 (22.9)
pT4	25 (10.8)
Nodal status	
N0	147 (63.7)
N1	65 (28.1)
Unknown	19 (8.2)
Metastases	
M0	203 (88)
M1	23 (10)
Unknown	5 (2)
Post-operative risk stratification for risk of recurrence (British Thyroid Association Guidelines 2014) [10]	
High	44 (19.1)
Intermediate	98 (42.4)
Low	82 (35.5)
Unknown	7 (3)
Surgery	
Nil	9 (4)
Lobectomy	31 (13)
Total thyroidectomy	161 (70)
Total thyroidectomy and neck dissection	30 (13)
^131^Iodine	
Nil	60 (26)
Ablation	171 (74)
^131^Iodine repeated	60 (26)
External beam radiation	
Neck, 56–66 Gy	9 (4)
Palliative	17 (7)
Nil	205 (89)
Recurrence	
Locoregional	21 (9.5)
Distant	2 (0.9)
Locoregional and distant	5 (2.2)
None	203 (88)
Deaths	
Total number of deaths	27 (11.6)
Deaths due to thyroid cancer	16 (6.9)

The median follow-up period was 57 months. Thirty patients were lost to follow-up and were censored at the last visit or last contact with the patient. The RFS at 10 years was 83%. The CSS and OS at 10 years was 91 and 84%, respectively. For patients who presented with metastases, the 10 year survival was 25%, with a median survival of 27 months.

Twenty-eight patients (12%) developed recurrence, with 21 having only loco-regional recurrence. Of the patients who had developed recurrence, 60% presented with clinically obvious recurrence, by way of enlarged neck nodes or bone pain. The remainder were detected by ultrasound of the neck or rising thyroglobulin levels. Surgery was performed for recurrent disease amenable to resection followed by ^131^I and external beam radiation, where indicated. Of the patients who had developed recurrence, 50% demonstrated no further evidence of disease following appropriate treatment.

The prognostic factors evaluated for RFS and CSS are shown in Tables 2 and 3, respectively. Univariate analysis demonstrated that male gender (*p* = 0.04), T4 (*p* = 0.038 for T1, *p* = 0.011 for T2), extra-thyroid extension (*p* = 0.02), nodal involvement (*p* = 0.004) and intermediate (*p* = 0.02), or high (*p* = 0.05) post-operative risk stratification, were significant for recurrence. However, on multivariate analysis (Table 4), only nodal disease (HR 4.523, *p* <  0.001) was independently predictive for recurrence.

**Table 2:** Univariate analyses for RFS

Variable	N (204)	10-year RFS %	*p*-value (log rank)	Prognostically unfavourable subgroup
Pathology				
Follicular and Hurthle	74	81	ns	–
Papillary	129	77		
Gender				
Male	34	78	0.04	Male
Female	170	81		
Age				
< 45	109	78	ns	
≥ 45	95	79		
T stage				
T0	1	100		
T1	49	85	0.038^Ϯ^	
T2	76	85	0.011*	T4
T3	48	75		
T4^Ϯ^*	16	60		
TX	14	63		
Extra-thyroid extension				
No	171	87	0.02*	Extra-thyroid
Yes*	23	64		extension
Unknown	10	53		
Nodal status				
N0^Ϯ^*	130	93		N1
N1	63	76	0.004*	and
NX (unknown)	11	30	< 0.001^Ϯ^	unknown
Residual tumour				
R0/1^Ϯ^	175	88		
R2^Ϯ^	15	100		Unknown
Unknown	14	24	< 0.001^Ϯ^	
Post-operative risk stratification for risk of recurrence				
High	17	72	0.05*	High, intermediate
Intermediate^Ϯ^	98	74	0.02*	and
Low*	82	90		unknown
Unknown	7	24	0.006^Ϯ^; 0.001*	

Symbols denote the associated prognostic variable for each *p*-value. Patients who presented with unresectable primary tumours and distant metastases were excluded (*n* = 27). These patients were not treated with curative intent and therefore could not be included in the recurrence free survival analysis

**Table 3:** Univariate analyses for CS survival

Variable	N	10-year CS survival %	*p*-value (log rank)	Prognostically unfavourable subgroup
Age				
Less than 25	27	100	0.01*	56–65
26–35	44	90	0.002*	
36–45	52	98	0.003*	
46–55	40	94	0.03*	
56–65*	41	72		
66–75	23	86		
Greater than 76	4	45		
Age				
< 45	116	97	< 0.001	≥ 45
≥ 45	115	80		
Gender				
Male	42	95	ns	–
Female	189	91		
T stage				
T0	1	100		T4
T1^Ϯ^*	53	98		and
T2^Ϯ^*	79	100		TX
T3^#^*	53	77		
T4	25	71	< 0.01^Ϯ^ 0.018^#^	
TX	20	65	< 0.01*	
Extra-thyroid extension				
No^Ϯ^	184	98	< 0.001*	Extra-thyroid
Yes*	31	76		extension and
Unknown	16	59	< 0.001 ^Ϯ^	unknown
Pathology				
Follicular and Hurthle	90	82		Follicular and
Papillary	140	95	0.012*	Hurthle
Nodal status				
N0^Ϯ^	147	96		NX
N1	67	89		
NX (unknown)	17	70	0.001^Ϯ^	
Residual tumour				
R0/1^Ϯ^	179	99		R2, n/a
R2	20	61	< 0.001^Ϯ^	and
n/a	18	56	< 0.001^Ϯ^	unknown
Unknown	14	85	< 0.001^Ϯ^	
Metastases				
M0	203	96	0.001*	M1
M1*	23	54		
MX (unknown)	5	67		
Post-operative risk stratification for risk of recurrence				
High*	44	46		
Intermediate	98	91	< 0.001*	High
Low	82	96	< 0.001*	
Unknown	7	80		

Symbols denote the associated prognostic variable for each *p*-value

**Table 4:** Multivariate analyses for RFS

Variable	Description	Parameter estimate	Standard error	*p*-value	HR (95% CI)
T stage	T4 vs T0–3	0.738	0.490	0.132	2.091 (0.800–5.464)
Extra-thyroid extension	Yes vs no	0.411	0.548	0.453	1.508 (0.515–4.415)
Gender	Male vs female	−0.700	0.425	0.100	0.496 (0.216–1.142)
Nodal status	Present vs none	1.509	0.408	< 0.001*	4.523 (2.032–10.070)

*denotes statistically significant *p* values

For CSS poor prognostic factors on univariate analysis were age ≥ 45 years (*p* <  0.001), T4 (*p* = 0.018 for T3, < 0.01 for T1 and T2), follicular pathological sub-type (*p* = 0.012),, extra-thyroid extension (*p* <  0.001), residual tumour (*p* <  0.001), metastases at presentation (*p* = 0.001) and high risk post-operative risk stratification for recurrence (*p* <  0.001). On multivariate analysis for CSS (Table 5) age ≥ 45 years (HR 22.746, *p* = 0.006), follicular pathology (HR 5.461, *p* = 0.004), extra-thyroid extension (HR 5.123, *p* = 0.013), and residual tumour (HR 24.219, *p* = 0.004) were independently predictive.

**Table 5:** Multivariate analyses for CSS

Variable	Description	Parameter estimate	Standard error	*p*-value	HR (95% CI)
T stage	T4 vs other	0.079	1.141	0.945	1.082 (0.116–10.119)
Metastatic disease at presentation	Present vs none	0.571	0.590	0.333	1.770 (0.557–5.628)
Extra-thyroid extension	Yes vs no	1.634	0.657	0.013*	5.123 (1.414–18.563)
Pathology	Follicular^a^ vs other	1.698	0.586	0.004*	5.461 (1.730–17.236)
Residual tumour	R2 vs other	3.187	1.115	0.004*	24.219 (2.721–215.549)
Age	< 45 vs ≥ 45	3.124	1.127	0.006*	22.746 (2.497–207.190)

*denotes statistically significant *p* values

^a^with Hurthle

Missing data groups that were found to be significant on univariate analysis for recurrence were nodal status (*p* <  0.001, NX vs N0), residual tumour i.e. no documentation on the surgical procedure was available indicating whether the tumour was completely resected or not (*p* <  0.001 for R0/1 and R2), and post-operative risk stratification for risk of recurrence (*p* = 0.001 for low risk and 0.006 for intermediate risk). For CSS, the significant factors were TX (*p* <  0.01 for T1, 2 and 3), nodal status (*p* = 0.001, NX vs N0), extra-thyroid extension (*p* <  0.001 for none), and residual tumour (*p* <  0.001 for R0/1).

Of the patients who had a total thyroidectomy and received ^131^I for ablation, 32 (18.7%) had persistent uptake of ^123^I on follow-up diagnostic scan and required further ^131^I administration. Nine patients had unresectable residual disease in the neck that was not iodine avid and were treated with external beam radiation to a dose of between 55 and 66 Gy in 2 to 2.4 Gy fractions. For the 23 patients presenting with metastatic disease, 18 (75%) had a total thyroidectomy followed by ^131^I.

## Discussion

This retrospective review showed that the known prognostic factors for differentiated thyroid carcinoma were relevant in a cohort of South African patients. In addition, the survival rates for our cohort of patients were similar to those observed at other centres. Our CSS was 94 and 91% at 5 and 10 years, respectively and the RFS was 91 and 83% for the same periods of observation. Analysis of the SEER database [16], reported a CSS of 98–99% at 5 years. Single institution reviews from Canada [4] and Germany [17] reported CSS rates at 10 years of 87.3 and 90.3%, respectively. For RFS, Mazzaferri et al. [6, 7] reported 30–35% recurrence rates with the majority occurring within the first 10 years after treatment.

Follicular carcinoma generally accounts for 15–24% of all differentiated thyroid carcinomas worldwide [4, 18–21]. However, we observed a higher proportion (35%) of follicular carcinoma in our patients. As mentioned previously, dietary deficiency of iodine appears to be associated with an increased risk of follicular carcinoma [22]. Prior to 1995, when legislation in South Africa required the addition of potassium iodate to table salt, a study assessing goiter prevalence and urine iodine concentration in children revealed evidence of iodine deficiency in the Mpumalanga, Northern Province and Eastern Cape provinces of South Africa [23]. Although the Western Cape was not included in the study, it is feasible that dietary deficiency may account for the higher percentage of follicular carcinomas in our patients.

We compared the tumour size and presence of cervical node metastases of our patient group at presentation with those described in retrospective reviews from North America. Reports on tumour size from a number of different studies showed a wide variation in tumour size. An analysis of relevant information from the SEER database from 1988 to 2009 by Krook et al. [16] indicated that 26.8% of patients had tumour sizes of < 1 cm and 11.98% of patients had tumour sizes of ≥4 cm. Investigators from Princess Margaret Hospital (PMH) reported that only 9% of patients had tumour sizes of < 1 cm and 29% had tumour sizes of > 4 cm [4]. A significant proportion of patients in both reviews and those in a study from Turkey [24] had tumour sizes ranging from 1 to 4 cm, which was consistent with the size range observed in our patients. Nodal metastases were present in 28.1% of our patients in comparison to other reports, in which it ranged from 10.5 to 50% [5, 16, 18, 19, 25]. The proportion of patients presenting with distant metastases was 10%, which is similar to the 9% reported from PMH [4], but higher than the 4% reported from the SEER database [5, 18].

There are several prognostic factors known to influence both recurrence and mortality in patients with differentiated thyroid carcinoma. The patient-related factors include age [4, 5, 7, 16, 17, 20, 25, 26] and gender, the latter being reported in a limited number of studies [4, 16, 25, 26]. The tumour-related factors include pathological sub-type, size of the primary tumour, lymph node metastases, extra thyroid extension, gross residual tumour and distant metastases [4, 5, 16, 17, 20, 25, 26]. These factors were reviewed in our cohort and, consistent with most other series, our study demonstrated similar prognostic factors for RFS and CSS on univariate analysis.

Groups characterised by missing data for certain tumour factors were assessed independently in the univariate analysis and significant associations with poorer prognosis were observed. Some undetermined factor may be responsible for the poorer prognosis however such associations may reflect inadequate management as a result of the limited information available for this group of patients. It is therefore important to ensure all prognostic variables are available and evaluated when making post-operative decisions.

For tumour recurrence the only factor that was independently predictive on multivariate analysis was the presence of nodal metastases. For mortality from thyroid carcinoma, significant factors found to be independently associated were age ≥ 45 years, follicular pathology, extra-thyroid extension and residual tumour. As stated previously, for the multivariate analysis, the groups of patients with missing data for each prognostic factor were included with the group that had a similar survival. In general, this was the prognostically unfavourable subgroup. Overall, distinct factors were found to affect recurrence and mortality.

Multiple studies have reported that the patient’s age at diagnosis affects prognosis [4–6, 16, 17, 20, 25, 26]. It is notable that differentiated thyroid carcinoma is the only cancer in the AJCC staging system in which age is incorporated into the stage. We followed the AJCC 6th and 7th editions, using 45 years as the cut-off age and found that there was a significantly higher risk of mortality from thyroid cancer for those ≥45 years [11, 12]. A recent analysis by Nixon et al. [27] of over 9000 patients, compared the disease specific survival for patients aged < 45 years with those aged < 55 years. The authors showed that 12% of patients could be down-staged and proposed that the cut-off age of 45 years be increased to 55 years for staging [27]. The recently published AJCC 8th edition has changed the age for poor prognosis from 45 to 55 years [28]. Age did not affect the risk of recurrence in our cohort.

Male patients have been reported to have a worse prognosis in some studies [4, 6, 16]. In our patients gender, although significant for recurrence on univariate analysis, was not significant for recurrence or survival on multivariate analysis.

Nodal involvement was a significant determinant of recurrence in our cohort. In the previous AJCC staging systems, the presence of pathological nodes affected the stage in patients aged 45 years and older with the presence of nodal metastases outside level VI, placing patients into the stage IV group. Patients aged less than 45 years, regardless of the presence of nodal metastases were all considered to be stage I. Brierly et al. [4] showed that the presence of nodal metastases significantly affect recurrence but not cause-specific survival. However, a publication by Adam et al. [9] reviewing the National Cancer Database (NCDB) and SEER has shown that nodal involvement, as well as the number of nodes involved, does affect survival in patients with papillary carcinoma who are younger than 45 years of age. Although the effect on overall survival at 10 years was relatively small, namely, 98.2% versus 97.8% for the NCBD and 98.7% versus 98.5% for SEER (HR for NCDB 1.32, *p* = 0.021, HR for SEER 1.29, *p* = 0.006), they suggested that, with the increasing incidence of papillary thyroid carcinoma in the USA*,* the absolute number of deaths could be significant [9].

Our patients with follicular carcinoma had a significantly higher risk of mortality than those with papillary carcinoma. This is consistent with reviews by Hundahl et al. [19], Mazzaferri et al. [7] and Shaha et al. [25]. In addition, extra-thyroid extension and gross residual tumour have been shown to affect prognosis [4, 18, 25, 26]. For our patients, none of these factors could be shown to predict for recurrence, but were significant for mortality. Since the approach to treatment was similar for the majority of patients we did not examine the effect of the extent of surgery or ^131^I ablation on recurrence or survival. Biological agents such as sorafenib and lenvatinib were not available for patients with iodine refractory disease.

Of the entire group, only 28 patients (12.5%) recurred and were treated with either surgery, 131-iodine, external beam radiation or a combination of these modalities. The median time to recurrence was 32 months although one patient developed recurrence 20 years after initial treatment. Notably, Mazzaferri et al. [7] reported recurrences in 30% of patients up to approximately 35 years after initial treatment. The majority, however, occurred within the first ten years and only 15% died after developing recurrence. Of our patients who developed recurrence, 50% had no evidence of disease following treatment for the recurrence. This outcome emphasises that patients require careful monitoring to detect early potentially curative recurrence.

The British Thyroid Association Guidelines published in 2002 [13] and updated in 2007 [14] recommended that all patients with a tumour size > 1 cm should be treated with total thyroidectomy, ^131^I ablation and TSH suppression. We followed these guidelines for this cohort of patients. In 2014 the guidelines were updated with identification of factors other than tumour size being taken into account to give a more personalised approach to patient management [10]. These updated guidelines have allowed a more conservative approach to surgery and ^131^I ablation in many of our current patients.

## Conclusions

In summary, we observed a higher proportion of patients with follicular carcinoma in our cohort when compared with other centres and this may be a result of iodine deficiency in the diet. The stage at presentation, prognostic factors, RFS and CSS were similar to reports from first world centres. Of the reported factors that affect prognosis in patients with differentiated thyroid cancer, only the presence of nodal metastases predicted for recurrence, while age ≥ 45 years, follicular pathology, extra-thyroid extension and residual tumour predicted for CSS. Our results demonstrate that, in a resource constrained setting, patients with differentiated thyroid carcinoma have a favourable prognosis with multidisciplinary team management and adherence to recognised treatment protocols. Since the current British Thyroid Association guidelines [10] allow for a less aggressive and more tailored approach to treatment it is important to continue to monitor our patients to ensure that similar outcomes are achieved.

## Abbreviations

^131^I: ^131^Iodine
AJCC: American Joint Committee on Cancer
CI: Confidence intervals
CSS: Cause specific survival
DTC: Differentiated thyroid carcinoma
ECOG: Eastern Cooperative Oncology Group
Gy: Gray
HR: Hazard ratio
IQR: Interquartile range
LR: Likelihood ratio
NCDB: National cancer database
OS: Overall survival
PMH: Princess Margaret Hospital
PS: Performance status
RFS: Recurrence free survival
SEER: Surveillance, Epidemiology, and End Results
SPSS: Statistical package for social sciences
T: Primary tumour
TSH: Thyroid stimulating hormone
USA: United States of America
